# Quercetin in the Prevention and Treatment of Coronavirus Infections: A Focus on SARS-CoV-2

**DOI:** 10.3390/ph15091049

**Published:** 2022-08-25

**Authors:** Amin Gasmi, Pavan Kumar Mujawdiya, Roman Lysiuk, Mariia Shanaida, Massimiliano Peana, Asma Gasmi Benahmed, Nataliya Beley, Nadiia Kovalska, Geir Bjørklund

**Affiliations:** 1 Société Francophone de Nutrithérapie et de Nutrigénétique Appliquée, 69100 Villeurbanne, France; 2 Birla Institute of Technology and Science-Pilani, Hyderabad 500078, India; 3 Department of Pharmacognosy and Botany, Danylo Halytsky Lviv National Medical University, 79010 Lviv, Ukraine; 4 CONEM Ukraine Life Science Research Group, Danylo Halytsky Lviv National Medical University, 79010 Lviv, Ukraine; 5 I. Horbachevsky Ternopil National Medical University, 46001 Ternopil, Ukraine; 6 Department of Chemical, Physics, Mathematics and Natural Sciences, University of Sassari, 07100 Sassari, Italy; 7 Académie Internationale de Médecine Dentaire Intégrative, 75000 Paris, France; 8 Bogomolets National Medical University, 01601 Kyiv, Ukraine; 9 Council for Nutritional and Environmental Medicine (CONEM), Toften 24, 8610 Mo i Rana, Norway

**Keywords:** quercetin, coronavirus, COVID-19, infection, SARS-CoV-2, nanopreparations, prevention, treatment

## Abstract

The COVID-19 outbreak seems to be the most dangerous challenge of the third millennium due to its highly contagious nature. Amongst natural molecules for COVID-19 treatment, the flavonoid molecule quercetin (QR) is currently considered one of the most promising. QR is an active agent against SARS and MERS due to its antimicrobial, antiviral, anti-inflammatory, antioxidant, and some other beneficial effects. QR may hold therapeutic potential against SARS-CoV-2 due to its inhibitory effects on several stages of the viral life cycle. In fact, QR inhibits viral entry, absorption, and penetration in the SARS-CoV virus, which might be at least partly explained by the ability of QR and its derivatives to inhibit 3-chymotrypsin-like protease (3CLpro) and papain-like protease (PLpro). QR is a potent immunomodulatory molecule due to its direct modulatory effects on several immune cells, cytokines, and other immune molecules. QR-based nanopreparations possess enhanced bioavailability and solubility in water. In this review, we discuss the prospects for the application of QR as a preventive and treatment agent for COVID-19. Given the multifactorial beneficial action of QR, it can be considered a very valid drug as a preventative, mitigating, and therapeutic agent of COVID-19 infection, especially in synergism with zinc, vitamins C, D, and E, and other polyphenols.

## 1. Introduction

Coronaviruses are positive-sense RNA viruses belonging to the family Coronaviridae and fall under the Nidovirales order. Since the beginning of the 21st century, coronaviruses have caused some of the most lethal outbreaks across the globe. For example, the SARS outbreak of 2002–2003 and Middle East respiratory syndrome coronavirus (MERS-CoV) outbreaks were caused by coronaviruses with a lethality rate of 10% and 37%, respectively [1]. A new member of the coronavirus family, SARS-CoV-2, is responsible for COVID-19, a highly contagious disorder that affects the respiratory system and leads to death in severe cases. Although the death rate of COVID-19 is much lower (~3.4%) than those of SARS and MERS, the highly contagious global outbreak has made COVID-19 one of the most lethal infections. The disease was first reported in Wuhan, China, in December 2019 and spread rapidly worldwide within months. COVID-19 has been declared a global pandemic by the WHO, and the number of coronavirus cases and the number of deaths continue to rise inexorably with a series of contagion waves [2,3,4]. Individuals infected with SARS-CoV-2 show pneumonia-like symptoms and develop a dry cough, intense fever, lung damage and inflammation, and breathing difficulty. In severe cases, lung damage is extensive and irreversible, leading to death. SARS-CoV-2 is a member of the β-coronavirus family and shares 79.5% sequence homology with the SARS-CoV virus (responsible for the SARS outbreak). SARS-CoV-2 also shares 96% sequence homology with bat SARS coronavirus, indicating that the novel coronavirus may have originated in bats. Once SARS-CoV-2 enters the body, the spike proteins of the virus interact with angiotensin-converting enzyme 2 (ACE2) receptors of the human alveolar epithelial cells. This interaction facilitates the entry of the virus into the host cells [5]. Studies have shown that SARS-CoV-2 is more lethal in patients with previous chronic disorders, such as diabetes, cardiovascular disorders, and lung diseases [5]. The current median incubation period of SARS-CoV-2 is 5.2 days (range: 0–24 days), and the median time between the symptoms and death is 14 days [5]. Despite the severity of the disease, no cure for COVID-19 is available. Some coronavirus proteins are essential to viral entry and replication; among them, the most attractive targets for drug development are papain-like protease (PLpro), 3C-like protease (3CLpro), and spike protein (S) [6].

Several scientific groups across the globe have started exploring various natural molecules for COVID-19 treatment. One such small molecule is quercetin (QR), a flavonoid molecule found in many natural products, such as vegetables, fruits, herbs, and bee products. QR, an antiviral agent, has been found effective in both SARS and MERS treatments. The present review is an attempt to understand various biological, pharmacological, and immunomodulatory properties of QR, which may be beneficial in the prevention and treatment of COVID-19. In addition, will be discussed the synergic effect of QR in combination with micronutrients, vitamins, trace elements, or other polyphenols.

## 2. Quercetin Sources and Properties

**Figure 1:**
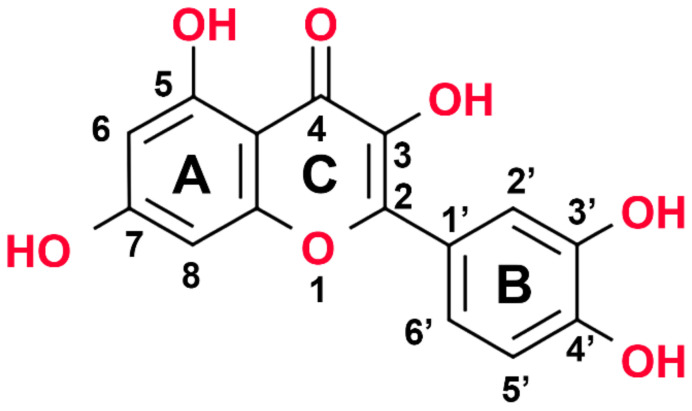
The structural formula of quercetin.

The increasing interest in recent years in naturally occurring plant phytochemicals for the healing of various diseases is because they are generally less expensive and have fewer side effects than synthetic drugs. QR and several other natural polyphenols act as antioxidants, scavengers of ROS and other free radicals, and induce phase II detoxification enzymes [7,8]. QR is a hydrophobic citron-yellow crystal and plant-derived substance that has been subject to experimental validation to evaluate its characteristics and biological properties [9]. QR is one of the most ubiquitous flavonoid molecules. The characteristic feature of QR is the presence of five hydroxyl groups at positions 3, 5, 7, 3′, and 4′ with the electron-donating activity (Figure 1).

QR possesses several biological effects, including antioxidant, anti-inflammatory, antiviral, anticarcinogenic, cardioprotective, psychostimulant, and neuroprotective properties [9].

Its ability to inhibit free radicals, the cause of oxidative stress, can decrease the risk of metabolic disorders, cardiovascular diseases, and certain types of cancer [8,10].

Some common food ingredients rich in QR are apples, berries, grapes, citrus fruits, tea, many seeds, nuts, honey, propolis, and medicinal plants [11,12,13,14]. High QR content was evaluated in some commonly eaten vegetables in Japan. During the acquisition period in the summer of 2013, it was found that the content of QR was 30.6 mg/100 g in red leaf lettuce (*Lactuca sativa* L. var. *crispa*), 23.6 mg/100 g in asparagus (*Asparagus officinalis* L.), 12.0 mg/100 g in romaine lettuce (*Lactuca sativa* L. var. *longifolia*), 11.0 mg/100 g in onion (*Allium cepa* L.), and 9.9 mg/100 g in green pepper (*Capsicum annuum* L.), and 2.1 mg/100 mL of QR in green tea infusion [15]. QR was the most abundant phenolic compound in acacia honey samples, ranging from 123.5 to 240.2 μg/100 g of honey [16].

This flavonol is widely distributed in plants, primarily as water-soluble QR glycosides [12]. The QR and derivatives are stable in the stomach of the human body under the gastric acid influence; glucosides are hydrolyzed in the small intestine by brush border enzymes, such as lactase phlorizin hydrolase, beta-glucosidase enzyme to the aglycone form, and then absorbed [17,18]. Thus, before absorption into the enterocyte, sugars must be removed from the molecule [18].

## 3. Antioxidant, Anti-Inflammatory, and Antitumor Activities of Quercetin

Various research groups have reported the pharmacological properties of QR, such as antioxidant, anti-inflammatory, and antitumor properties. Due to these properties, QR is recommended for managing various disorders where oxidative stress, inflammation, and abnormal cell proliferation are major underlying causes. Zhang et al. observed that QR has a higher reduction potential than curcumin, comparable to the standard antioxidant Trolox. Moreover, QR reduced lipopolysaccharide (LPS)-induced production of reactive oxygen species and nitric oxide levels. The data indicated that QR is a powerful antioxidant and anti-inflammatory agent [14,19]. QR also increased the oxidative stress-fighting ability of the cells by stimulating the synthesis and expression of antioxidant enzymes, such as catalase, glutathione peroxidase, and superoxide dismutase. These enzymes’ QR-induced expression protects the tissues from oxidative damage and injury [20]. Oxidative stress and inflammation are interlinked in the way that the presence of one of these phenomena induces the appearance of the other, and both are commonly observed in several chronic disorders, such as obesity, type 2 diabetes mellitus (T2DM), and cardiovascular disorders (CVDs) [21]. This indicates that reducing oxidative stress/inflammation profoundly alleviates the symptoms of chronic diseases, and consequently, QR can be used as a powerful therapeutic strategy to treat these chronic disorders [21]. QR inhibits inflammation by reducing the expression of the cyclooxygenase (COX) and lipoxygenase (LOX) enzymes. The inhibition of these enzymes by QR reduces the synthesis of leukotrienes and prostaglandins, critical mediators of inflammation in the body [22,23]. Another key marker of inflammation in the body is C-reactive protein (CRP), and elevated levels of CRP have been implicated in several disorders, such as obesity, T2DM, and CVDs. QR inhibits the levels of several proinflammatory molecules, such as nitric oxide, COX, and CRP in hepatocyte cell lines [24].

Moreover, in [25], a dose of 80 mg significantly reduced chronic inflammation and helped in cases of adjuvant-induced arthritis. QR is also a potent anticancer agent by promoting apoptosis in cancer cells (CT-26, LNCaP, MOLT-4, and Raji cell lines) and reducing the volume of tumors [26]. QR inhibits cancer cell proliferation, reduces neovascularization of tumors, induces apoptosis, and prevents tumor metastasis [27]. QR (100 µM) causes cell growth inhibition and halts the proliferation of human T leukemic lymphoblasts (Jurkat) [28].

Considering its potent antioxidant properties, QR is actively investigated as a promising substance for the prevention and treatment of CVDs [29]. QR contributes to a decreased incidence of stroke due to radioprotective characteristics mediated by its impact on proteasomal proteolysis, as demonstrated in an experimental model of cholesterol-induced atherosclerosis in rabbits. One-month administration of QR decreased atherosclerotic lesion areas in the aorta [29]. In the same animal model, the QR derivative corvitin suppressed lipid peroxidation [30]. QR exhibited an antioxidant effect and a positive impact on endothelial function in patients with acute coronary syndrome with ST-segment elevation [31]. Treatment with QR-containing medicines positively affected hemodynamics and decreased about threefold the cardiac fibrosis area [32]. The anti-ischemic activity of intravenous QR in patients with ST-segment elevation myocardial infarction has been demonstrated [33].

## 4. Quercetin in Immunomodulation

QR is a potent immunomodulatory molecule due to its direct modulatory effects on several immune cells, cytokines, and other immune molecules. For instance, QR treatment reduces the expression of major histocompatibility complex (MHC) class II and other molecules that stimulate dendritic cells (DCs). DCs isolated from mouse bone marrow display reduced activation when administered with LPS. QR also decreases the LPS-induced migration of DCs. The reduction of DC activation reduces the antigen-specific activation of T-cells in the body [34,35]. The action of QR on immunity and inflammation is carried out by acting primarily on leukocytes and targeting different intracellular signaling kinases and phosphatases, enzymes, and membrane proteins [36].

Flavonoids exhibit numerous biological effects. In addition to antioxidant, anti-inflammatory, and antiviral properties, hepatoprotective and antiallergic properties have also been demonstrated [37]. The “cytokine storm”, an increased release of tumor necrosis factor (TNF), interferon-gamma (IFN-γ), interleukins, and other cytokines, is one of the main characteristics of severe viral infections, including SARS and COVID-19 disease [1]. Flavonoids are considered promising anti-inflammatory therapeutic agents due to several studies on their in vitro inhibitory activity towards various inflammatory mediators, including cytokines [36]. QR shows potent immunomodulatory properties by inhibiting the expression of several proinflammatory cytokines and signaling pathways. Rogerio et al. demonstrated that treatment with QR-loaded microemulsion (QR-ME) (3 or 10 mg/kg) during a 22-day study reduced airway inflammation by decreasing the expression of IL-5 and IL-4. QR-ME also reduced the activation of the NF-κB inflammatory pathway and the expression levels of P-selectins [38]. QR (20 mg/kg/day, intraperitoneally) decreased hyperoxic lung injury in neonatal mice by reducing inflammation and enhancing alveolarization with an impaired degree of neutrophil and macrophage infiltration [39]. Another study by Stemberg et al. showed that QR modulates the activity of peripheral blood mononuclear cells (PBMCs) isolated from multiple sclerosis patients. QR treatment significantly reduced the PBMC proliferation dose-dependently and consequently reduced the expression levels of TNF-α and IL-1β. The modulating effect was more pronounced when QR was given in combination with IFN-β [40]. QR treatment also modulates the activity of cytokines, such as IL-4 and IL-5 secreted by Th2 cells.

Moreover, a reduction in specific immunoglobulin E (sIgE) levels was observed, leading to a reduced anaphylactic reaction. These immunomodulatory properties of QR are useful in alleviating asthma symptoms [41]. QR treatment in immunized mice increased B-cell proliferation under ex vivo conditions and enhanced the numbers of IgM-producing lymphocytes [42]. The immunomodulatory properties of QR were also evident in inflammatory responses induced by the influenza A virus. In such cases, QR treatment increased the secretion of IL-27 and reduced TNF-α expression [43]. A study by Zhang et al. demonstrated that QR possesses antifatigue properties, which is attributed to reduced TNFα expression and increased the expression of IL-10 in a strenuous exercise mice model [44]. The intake of QR is also beneficial in treating food allergy induced by peanuts by reducing the expression of immunoglobulin E responses [45]. QR also modulates the immune system by decreasing the expression of IL-4, inhibiting the activity of eosinophils, improving Th1/Th2 balance, and reducing the levels of leukotrienes and prostaglandins [45].

## 5. Antimicrobial Activity of Quercetin

QR has been studied for its antimicrobial actions, and several studies have shown that QR inhibits the growth of several bacterial strains. For example, Jaisinghani et al. reported that QR inhibits the growth of several bacterial strains, such as *Staphylococcus aureus*, *Proteus vulgaris*, *Shigella flexneri*, *Escherichia coli*, and *Pseudomonas aeruginosa*. However, the inhibitory concentration of QR varied, which was 20 μg/mL for *S. aureus* and *P. aeruginosa*. Other bacterial strains were inhibited at a higher concentration of QR: *P. vulgaris* (300 μ/mL), *E. coli* (400 μg/mL), *S. flexneri* (500 μg/mL), and *Lactobacillus casei* (500 μg/mL) [46]. In another observation, Wang et al. demonstrated its bacteriostatic action in *E. coli*, *S. aureus*, *P. aeruginosa*, and *Salmonella enterica Typhimurium* species. QR was more effective in inhibiting the growth of Gram-positive bacterial species than Gram-negative bacteria. At the molecular level, QR inhibits the growth of *E. coli* and *S. aureus* by damaging the cell wall and cell membrane. This increased cell wall/membrane damage is reflected in the increased activity of extracellular enzymes, such as alkaline phosphatase and β-galactosidase [47]. QR also demonstrated antibacterial action against periodontal pathogens, such as *Actinobacillus actinomycetemcomitans* (Aa) and *Porphyromonas gingivalis* (Pg). A study by Geoghegan et al. showed that QR inhibits the growth of Aa and Pg in a dose-dependent manner and hence shows promise in preventing bone loss. However, Aa showed growth inhibition only up to 12 h, followed by which the antibacterial action of QR decreased. However, Pg showed growth inhibition after 24 h of treatment [48]. In an interesting observation, Siriwong et al. showed the antibacterial action of QR against the multidrug-resistant strain of *Staphylococcus epidermidis.* The strain used in the study was amoxicillin-resistant *S. epidermidis* (ARSE). It was observed that a combination of QR and amoxicillin showed synergistic effects on bacterial growth inhibition. The combination promoted cell membrane permeability, induced damage to the cytoplasmic membrane, reduced the bacterial cells’ fatty acid content, and inhibited peptidoglycan synthesis. QR also inhibited the activity of β-lactamase, the principal enzyme involved in the degradation of β-lactam antibiotics [49]. Hirai et al. reported that QR showed antibacterial action against methicillin-resistant *S. aureus*. Moreover, QR showed synergistic activity against MRSA when combined with other antibiotics, such as erythromycin, vancomycin, ampicillin, oxacillin, and gentamicin. QR also induced the aggregation of *S. aureus* cells and affected the colony-spreading ability of *S. aureus* [50].

## 6. Antiviral Properties of Quercetin

Targeting viral proteins responsible for viral entry into the host cells can be an important target for inhibiting viral infections. QR has shown antiviral properties by inhibiting the entry of viruses into the host cells. For example, QR inhibited the initial stages of virus infection in several strains of influenza virus, such as A/Puerto Rico/8/34 (H1N1), A/FM-1/47/1 (H1N1), and A/Aichi/2/68 (H3N2). It was observed that QR interacts with hemagglutinin (HA2 subunit), a glycoprotein responsible for the entry of the virus, and prevents viral entry [51]. Another way to inhibit or slow down virus infection is to target various stages of the virus life cycle, such as the assembly and release of mature virus particles from the cells. It was shown that QR reduced the copy numbers of NP and M2 proteins, which code for influenza viral nucleoproteins and channel proteins, respectively [43,52]. QR also prevented virus-induced cellular apoptosis and reduced virus progeny yields.

Moreover, QR was also effective in inhibiting postattachment stages of viral infection [53]. In a mouse model of chronic obstructive pulmonary disease caused by rhinovirus, QR inhibited viral replication and reduced lung inflammation. QR also decreased the mRNA levels of types I and II interferons and IL-13 while increasing the expression of IL-10. The authors concluded that QR could be used to improve lung performance and reduce various symptoms of respiratory distress. Since COVID-19 patients also show severe lung inflammation and breathing difficulty, QR can be a potential agent to reduce health complications [54]. QR also stimulates the antiviral signaling of mitochondria, improving the body’s disease-fighting ability [54]. QR prevented Ebola infections when injected into mice 30 min before infection, inhibiting the early stages of the Ebola virus infection [55]. A natural QR derivative, quercetin-3-β-O-d-glucoside, reduced Zika infections in nonprimates [56].

## 7. Quercetin as a Preventive and Therapeutic Agent for COVID-19

QR is an active agent against the SARS virus, and SARS-CoV-2 shares a close resemblance to it. QR could hold therapeutic potential against SARS-CoV-2 due to its inhibitory effects on several stages of the viral life cycle [57,58,59,60,61,62,63]. In particular, QR can alter, in human cells, the expression of 30% of genes encoding protein targets of SARS-CoV-2, thus potentially interfering with the activities of 85% of these proteins [62]. Potential drug targets in the virus are PLpro, 3CLpro, RNA-dependent RNA polymerase (RdRp), and viral spike glycoproteins. In contrast, drug targets presented on the host are angiotensin-converting enzyme (ACE2), angiotensin AT2 receptor, and transmembrane protease serine 2 (TMPRSS2) [64].

The inhibition of the interaction between the glycoprotein spike of SARS-CoV-2 and ACE2 is a potential antiviral therapeutic approach. QR is a potent inhibitor of recombinant hACE2 at physiologically relevant concentrations in vitro, with an IC_50_ of 4.48 μM [65]. Small molecules that bind with the surface spike proteins of SARS could prevent the SARS virus’s entry into the host cells. Yi et al. demonstrated that QR showed antiviral activity against HIV-luc/SARS (EC_50_: 83.4 μM) by inhibiting its entry. Moreover, QR displayed very low cytotoxicity against normal cells, thus making it a potential small molecule for treating the SARS virus [66]. QR inhibits viral replication by inhibiting the activity of the SARS virus. The 3-chymotrypsin-like protease (3CLpro), required for SARS-CoV replication, has been proposed as a potential drug target for SARS. Past studies by Chen et al. demonstrated inhibitory activities of QR derivatives on 3CLpro of SARS-CoV [67]. Nguyen et al. demonstrated that QR inhibits 3CLpro, expressed in *Pichia pastoris*, with an IC_50_ value of 73 μM [68]. It is relevant to note that 3CLpro, also called the main protease (Mpro), is a potential drug target for COVID-19, and Zhang et al. recently crystallized 3CLpro of SARS-CoV-2 with an α-ketoamide inhibitor [69]. A recent molecular docking study by Khaerunnisa et al. showed that QR binds with 3CLpro of SARS-CoV-2 with a binding energy of −8.58 kcal/mol [70]. In another recent molecular docking study, the QR derivatives quercetin 3-β-d-glucoside and quercetin 3-d-galactoside showed some potential against SARS-CoV-2 [71]. S1 and S2 of 3CLpro of MERS-CoV sites play an important role in the interaction with flavonoids. Some QR derivatives with hydrophobic nature and attached carbohydrate moieties could be potential drug targets for 3CLpro inhibition. Jo et al. used a flavonoid library to find inhibitory compounds against 3CLpro of MERS-CoV, a coronavirus with a very high mortality rate (~35%). It was observed that the QR derivative quercetin-3-β-d-glucoside blocked the activity of 3CLpro. The binding between the QR derivative and the enzyme was confirmed using the tryptophan-based fluorescence method [72]. Another molecular docking study indicated that QR might be an inhibitor of the Mpro protein (6flu7) of SARS-CoV-2. The binding affinity of ligands by QR was −7.1 Kcal/mol [73].

The inhibitory potency of quercetin against the RNA-dependent RNA polymerase (RdRp), responsible for viral genome replication, was evaluated by De Vivo et al. The IC_50_ value in the biochemical enzymatic assay was 6.9 ± 1.0 µM [74]. Thus, also RdRp could be pharmacologically targeted for SARS-CoV-2 replication inhibition.

## 8. Quercetin—Micronutrients Potential Synergies in the Treatment of COVID-19

Several polyphenols, flavonoids, vitamins, and minerals/trace elements are beneficial in fighting viral disorders due to their multiple physiological and biological effects [75]. For example, vitamins are known to boost immunity, enhance the immune response against the virus, and improve the biological activity of phytoconstituents (including flavonoids). Similarly, flavonoids reduce oxidative stress, inflammation, and the disease-fighting ability of the system [76]. Thus, it is expected that a combination of QR, a flavonoid, and other polyphenols and vitamins shows a synergistic effect and helps the speedy clearance of the virus. The combination of QR and other polyphenols is a strategy to control the viral infection by attacking several targets simultaneously [77]. This combination also helps reduce the doses of flavonoids and polyphenols, thus diminishing the development of viruses resistant to drugs/natural compounds [77]. For example, a recent study by Aslam et al. demonstrated that a combination of various herbal extracts rich in flavonoids and polyphenols showed synergism and displayed improved antioxidant capacity and free radical scavenging potential [78].

Moreover, a combination of plants has been reported to treat viral infections causing respiratory tract, throat, skin, and nasal cavity [79]. Taken together, using QR in combination with other polyphenols and vitamins can be a better therapeutic choice than using them individually [75,80]. Using a specific and well-designed combination of polyphenols has the advantage of a high safety profile without causing significant side effects [81].

## 9. Quercetin in Combination with Other Polyphenols

Since SARS-CoV-2 is a novel coronavirus, no study reports a synergistic effect of QR with other polyphenols. However, the synergistic antiviral properties of QR are well documented in previous studies on other viruses. For example, QR synergistically affected herpes simplex virus type 1. In this study, a combination of QR (0.4 mM) and apigenin (0.4 mM) showed synergistic behavior in clearing the virus in the cell culture. It is important to highlight that synergistic action is observed only when both molecules act on different targets to inhibit virus growth. This leads to the inhibition of multiple molecular pathways or concurrent inhibition of targets leading to synergistic behavior [82]. In another study, a combination of QR, naringenin, and pinocembrin showed a synergistic effect in inhibiting canine distemper virus. The study reported that combining QR, naringenin, and pinocembrin before viral infection enhanced cell survival and inhibited CDV-NP gene expression [83]. Chiow et al. reported that a combination of QR and quercitrin at a 1:1 ratio displayed increased inhibition of the virus and lower cell cytotoxicity than using them individually. The study reported the synergistic antiviral behavior of QR in the DENV-2 virus [84]. A recent report on COVID-19 recommended several traditional Chinese medicinal plants/formulations where QR is one of the major constituents of the proposed formulation. As per the report, 26 herbs cataloged for treating respiratory infections (caused by viruses) may contain QR in combination with other flavonoids and polyphenols. For example, Shufeng Jiedu Capsule (SFJDC), a well-known formulation in traditional Chinese medicine (TCM), contains QR in combination with other polyphenols and flavonoids, such as rutin, kaempferol, liquiritigenin, liquiritin, resveratrol, emodin, and rhein. SFJDC is one of the recommended TCM formulations for the treatment of influenza in China [85].

Zhang et al. [6] conducted in silico screening of 125 species of TCM and found that several phenolics (QR, kaempferol, and lignin) may inhibit COVID-19 reproduction. Among the investigated constituents of 121 herbs in a study, luteolin and tetra-O-galloyl-β-d-glucose inhibited SARS-CoV entry into a host cell [66].

After the screening of thousands of potential antiviral phytoconstituents in a medicinal plant database, the following were found to be the most promising polyphenols for inhibiting SARS-CoV-2 3CL virus replication [86]: myricitrin, myricetin 3-O-beta-D-glucopyranoside, methyl rosmarinate, flavanone-3-O-beta-D-glucopyranoside, 5,7,3′,4′-tetrahydroxy-2′-(3,3-dimethylallyl) isoflavone, (2S)-eriodictyol 7-O-(6″-O-galloyl)-beta-d-glucopyranoside, and calceolarioside B. A phenolic aloe-emodin compound, isolated from *Isatis indigotica* root, dose-dependently inhibited the 3CLpro cleavage activity (IC_50_ = 366 mM) [87]. Yu et al. found in vitro that the flavonoids myricetin and scutellarein can inhibit the SARS-CoV helicase protein [88]. Glycosides of the flavonol kaempferol may be good antiviral agents for 3a channel proteins of coronaviruses [89].

The geranylated flavonoids tomentins (A, B, C, D, and E), isolated from the *Paulownia tomentosa* fruit extract, inhibited the papain-like protease of SARS-CoV [90]. Isobavachalcone and psoralidin in *Psoralea corylifolia* seeds also decrease the SARS-CoV papain-like protease activity [91]. Papyriflavonol from *Broussonetia papyrifera* extract was, in a study, the most potent inhibitor of papain-like coronavirus cysteine proteases (IC_50_ = 3.7 μM) [92].

*Cinnamomi cortex* butanol extract demonstrated anti-SARS-CoV activities through several mechanisms due to the yield of different compounds or mixtures. Still, procyanidins were evaluated as the main constituents with such properties [93]. Unique compounds found among the green tea polyphenols were epigallocatechin-3-gallate and its lipophilic derivatives [94]. Fatty acid monoesters of epigallocatechin-3-*O*-gallate showed an antiviral effect against several viruses, probably due to their affinity for viruses and cellular membranes [95]. Investigation of the 3CL(Pro) inhibitory effect of extracts from seven different teas revealed that the polyphenol theaflavin-3,3′-digallate possesses this activity [96]. Besides QR, such flavonoids as luteolin, apigenin, kaempferol, amentoflavone, epigallocatechin, gallocatechin gallate, and epigallocatechin gallate were found as efficient blockers of the SARS-CoV 3CLpro enzymatic activity [97]. Ellagic acid proved to be the most potent 3CLpro inhibitor among numerous polyphenols tested by in silico molecular docking and molecular dynamics supported by in vitro assays [98]. According to Ryu et al. [99], after the fractionation of the *Torreya nucifera* leaf extract, a good SARS-CoV 3CL inhibitory activity was revealed by the biflavone amentoflavone (IC_50_ = 8.3 μM).

It has been observed that seriously ill patients with COVID-19 show pulmonary inflammation, and the pulmonary inflammation load score is higher in patients with an advanced stage of COVID-19 than in patients with a mild condition. Thus, reducing inflammation is a critical target for reducing the severity of COVID-19 [100]. A study by Heeba et al. demonstrated that a combination of QR and curcumin (both 50 mg/kg) showed synergistic behavior in reducing inflammation and oxidative stress in a rat model. The mixture was more effective in reducing inflammatory cytokine TNF-α, MDA (an indication of lipid peroxidation), and nitric oxide levels. Moreover, the combination increased heme oxygenase-1 levels and restored the levels of GSH, indicating reduced oxidative stress [101].

## 10. Quercetin in Combination with Vitamins and Trace Elements

Vitamins are essential for several physiological processes in the system and boost immunity. Using vitamins in combination with QR can alleviate the symptoms of COVID-19. Vitamin C is a potent antioxidant molecule and could be beneficial in reducing the symptoms of coronavirus. Several recent clinical trials on COVID-19 used vitamin C to treat infected patients. The RTC NCT04468139 is based on an approach of quadruple therapy including QR, vitamin C, zinc, and bromelain (https://clinicaltrials.gov/ct2/show/NCT04468139, accessed on 29 July 2022).

It has been reported that a higher level of intracellular zinc increases intracellular pH inhibiting SARS-CoV-2′s RNA-dependent RNA polymerase, leading to damage to the virus replication mechanism [102]. QR as zinc ionophore helps increase intracellular zinc influx. The dietary supplement bromelain (a proteolytic enzyme from the pineapple plant) has been shown to diminish the expression of ACE2 and TMPRSS2 and to inhibit SARS-CoV-2 infection in Vero E6 cells [103]. In COVID-19, SARS-CoV-2-induced sepsis leads to a surge in the levels of proinflammatory cytokines, leading to increased accumulation of neutrophils in the lungs and further destruction of alveolar capillaries. Vitamin C cannot only prevent this accumulation, but it also does alveolar fluid.

Moreover, vitamin C prevents vascular injury by inhibiting the formation of neutrophil extracellular traps. Vitamin C also averts the common cold and protects against influenza [104]. Since COVID-19 patients show fever, cough, inflammation, and respiratory distress, a combination of vitamin C and QR can be useful in treating the disorder because both are potent antioxidant and anti-inflammatory molecules. Although QR is a ubiquitous flavonoid, and its dietary intake can be easily enhanced by changing nutritional habits, its lower absorption rate in the gastrointestinal tract limits its therapeutic effects. Normally, the absorption rate of QR in the intestine is between 30% and 50%, but a higher half-life of 25 h can help maintain the plasma levels of QR. It has been observed that vitamin C increases the absorption rate of QR in the intestine and elevates plasma QR levels [105].

Vitamin C also reduces flavonol’s oxidative degradation, thus helping maintain higher plasma levels of flavonoids, such as QR [105]. Vitamin D is a major immunomodulatory vitamin and controls the immune system. Studies have established that vitamin D prevents respiratory distress by regulating the activity of the immune system and eliminating viral pathogens. It downregulates excessive cytokine secretion during viral infection and helps clear the pathogen [106]. These properties of vitamin D can be beneficial in combination with QR. In addition, QR shows synergistic behavior with vitamin E and protects against oxidative damage. The combination of QR and vitamin E reduces free radical damage and augments cellular defense against ROS [107]. In another observation, QR and vitamin E combination significantly reduced metal intoxication, particularly towards the nonessential cadmium [108,109]. Several studies have shown that combining QR and vitamins can synergistically increase the antioxidant capacity, reduce inflammation, and eliminate viral pathogens. These properties are beneficial in treating COVID-19, where oxidative stress, respiratory distress, and inflammation are important symptoms. However, further research in this direction is needed to explore the appropriate combinations and ratios of QR and vitamins.

## 11. Quercetin-Based Nanopreparations

Nanotechnology is a modern and promising area of science and technology that allows solving several problems in many fields, including medicine. The reduction of the particle sizes in the nanometer scale enhances their solubility, activity, and bioavailability [110,111]. Nanomaterials improve active principles’ physical, chemical, and biological characteristics [112].

Due to poor bioavailability (low aqueous solubility and permeability) and instability in physiological media of QR, high doses of this substance are required for administration, which is the main limitation of its clinical application.

QR-based nanosystems, reducing hydrophobicity and increasing the bioavailability of the active ingredient, can have promising proficiency as antiviral agents. Several scientific studies apply various techniques to obtain QR formulations with enhanced bioavailability and water solubility [113,114,115,116,117,118,119]. A chemically and photostable QR nanosuspension with a significantly increased dissolution level was obtained by Gao and colleagues [113]. Poly(lactic-co-glycolic acid) nanoparticles loaded with QR were successfully tested on the human triple-negative breast (MDA-MB-231) and larynx epidermoid carcinoma (HEp-2) cell lines, revealing potent antiproliferative and cytotoxic effects on cancer cell lines [115]. Encapsulated in solid–liquid, QR exhibited a sustained QR release until 48 h and higher efficacy in inhibiting MCF-7 human breast cancer cells [118]. QR nanoparticles revealed activity towards mechanisms involved in amyloid-related diseases [116]. A more soluble and safe food-grade formulation of QR based on lecithin (quercetin phytosome) was recently developed. In comparison with QR alone, quercetin phytosome showed improved oral absorption with the detection of 20-fold exceeded QR in the plasma [119]. Several studies have reported that patients treated with QR phytosome supplementation have better clinical outcomes than those treated with standard therapy [120].

## 12. Conclusions

COVID-19 is a severe respiratory disorder caused by SARS-CoV-2. The disease is highly contagious and spreads quickly in the community through contagion waves. Since no cure for COVID-19 is available, several scientific groups worldwide have shifted their focus to finding treatment from natural sources. Natural substances have been found effective in treating SARS and MERS due to their inhibitory effects on virus entry, absorption, penetration, and replication. Quercetin, a flavonoid naturally occurring in fruits, vegetables, tea, medicinal plants, and bee products, is a potent antiviral drug molecule against SARS and MERS.

**Figure 2:**
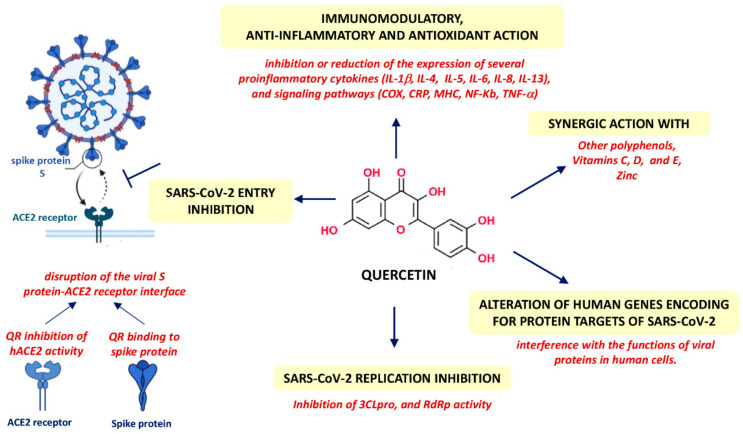
Beneficial multifactorial action of quercetin as a preventive, mitigative, and therapeutic agent of COVID-19 infection.

Consequently, it has been proposed as possibly useful for the COVID-19 cure. The potential beneficial effect of quercetin in the treatment of COVID-19 has been evaluated in recent case-control clinical studies that found its efficacy in inhibiting SARS-CoV-2. Quercetin shows multifactor beneficial action against SARS-CoV-2 to counterbalance the COVID-19 infection (Figure 2).

Quercetin showed inhibitory effects on several stages of the viral life cycle, from entry to replication. In particular, quercetin could directly bind the glycoprotein spike and inhibit the activity of ACE2, thus disrupting the viral–host recognition interface and preventing the SARS-CoV-2 entry. Quercetin can alter the expression of several human genes encoding protein targets of SARS-CoV-2, thus potentially interfering with the functions of the viral proteins in human cells. Quercetin inhibits viral replication by interfering with the activity of 3-chymotrypsin-like protease (3CLpro), papain-like protease (PLpro), and RNA-dependent RNA polymerase (RdRp). Moreover, quercetin possesses a wide spectrum of antioxidant, anti-inflammatory, and immunomodulation actions contributing to mitigating the disease consequences.

Besides quercetin, several phenolic compounds are prospective for treating COVID-19 infection. The efficacy of quercetin can be amplified with the synergism with these polyphenols and with the beneficial action of vitamins C, D, and E and zinc. Several clinical trials related to the monotherapy of quercetin and their compositions, including zinc, vitamin C, curcumin, vitamin D3, and drugs such as hydroxychloroquine, azithromycin, masitinib, and ivermectin, have been launched. Among the available results, the confirmation of the efficacy of some combinations tested for the prevention of COVID-19 is evident [121].

However, large well-designed RCTs of quercetin-based compositions are still needed to identify an effective COVID-19 treatment, considering the emerging SARS-CoV-2 variants. Further research will need to improve the bioavailability and solubility of quercetin and its drug combinations to improve the absorption rate.

Nanopreparations appear to be among the most promising solutions.

## Data Availability

Data sharing not applicable.
